# Paternal drinking in western China is associated with preschool children’s well-being: a cross-sectional SDQ study

**DOI:** 10.3389/fpsyt.2026.1739728

**Published:** 2026-04-10

**Authors:** Minna Liu, Hongli Sun, Xi Zhang, Wei He

**Affiliations:** 1 Child Healthcare Department, Northwest Women and Children’s Hospital, Xi’an, China; 2 Shaanxi Institute for Pediatric Diseases, Xi'an Key Laboratory of Children's Health and Diseases, Xi'an Children's Hospital (Affiliated Children's Hospital of Xi'an Jiaotong University), Xi’an, China; 3 Department of Clinical Laboratory, Xi'an Children's Hospital, Affiliated Children's Hospital of Xi'an Jiaotong University, National Regional Children's Medical Center (Northwest), Xi'an, China

**Keywords:** mental health, paternal alcohol, preschoolers, SDQ, western China

## Abstract

**Background:**

Limited evidence links paternal drinking (41.5% prevalence) to preschoolers’ behavioral difficulties in Western China, necessitating Strengths and Difficulties Questionnaire-based studies to assess psychosocial outcomes.

**Method:**

From February 28 to March 5, 2025, a cross-sectional study enrolled 25,017 parent-child dyads across 189 public kindergartens in a western city, China, using stratified sampling. After excluding 2,408 non-parents and 1,397 dyads with invalid age data, 21,212 remained. Paternal alcohol intake was categorized as nondrinkers, ex-drinkers, or current drinkers (light [≤ 2.86 g/day], moderate [>2.86-20 g/day], heavy [>20-40 g/day]. Child mental health was assessed using the Strengths and Difficulties Questionnaire (SDQ), with total difficulties (TDS >14 vs. ≤14) and prosocial behavior (PB <6 vs. ≥6) as categorical outcomes. Multivariable logistic regression, including a combined model of drinking status and daily intake, examined associations with these outcomes, adjusting for child, parental, and household factors. Continuous scores (TDS, PB, and SDQ subscales) were analyzed using multivariable linear regression, with false discovery rate correction applied to subscale analyses. Stratified analyses tested effect modification by covariates.

**Results:**

Paternal alcohol consumption was consistently associated with adverse child mental health outcomes. Ex−drinkers had elevated odds for both total difficulties (TDS >14; adjusted OR = 1.54, P <0.001) and reduced prosocial behavior (PB <6; adjusted OR = 1.43, P <0.001). Among current drinkers, dose-response patterns were evident: for TDS, odds rose from 1.21 for light drinking to 1.73 for heavy drinking, with each additional 100 g/week associated with 20% higher odds (P <0.001); For PB, the association with paternal alcohol intake was weak but statistically significant (OR = 1.01, p = 0.01), reflecting a flat trend without clear dose-response. Stratified analyses revealed stronger associations for TDS among boys, higher−income households, and children of non−working fathers; paternal effects were attenuated when mothers were current drinkers; for PB, maternal employment status was the sole significant modifier.

**Conclusions:**

Paternal current and ex−drinking were associated with adverse child mental health outcomes. Future longitudinal studies with interaction models and larger samples are needed to confirm mechanisms and refine high−risk subgroup identification.

## Introduction

Childhood mental health—encompassing emotional regulation, behavioral adjustment, and prosocial development—is a critical global public health priority (1). Early-onset behavioral and emotional difficulties predict long-term adverse outcomes, including academic underachievement and psychiatric disorders in later life (2, 3), underscoring the importance of identifying modifiable early-life risk factors (4).

The family environment plays a pivotal role in shaping early childhood development (5). While maternal influences have been extensively studied (6, 7), paternal factors remain comparatively understudied despite growing evidence that fathers’ behaviors and wellbeing significantly affect child outcomes (8, 9). Paternal alcohol consumption has emerged as a relevant risk factor, with Western studies demonstrating associations with childhood behavioral and emotional problems (10–12). Proposed mechanisms include family conflict, compromised parenting, and economic strain, with heavier drinking conferring greater risk (13).

Extensive evidence demonstrates that alcohol use is transmitted across generations. Family, twin, and adoption studies show that children of parents with alcohol use disorders have a two- to fourfold increased risk of developing alcohol problems, with heritability estimates of 50-60% (14–17). These risks reflect genetic liability (e.g., reward processing, inhibitory control (18)) and gene-environment interplay (19). Importantly, parental alcohol misuse is also associated with elevated internalizing and externalizing problems in children (20, 21), and paternal drinking specifically predicts children’s psychosocial difficulties even after accounting for maternal drinking and broader family adversity (22, 23).

Multiple mechanisms may underlie these associations. Through social learning, children may normalize paternal drinking and model dysregulated behaviors (24, 25), while paternal heavy drinking is linked to conflict, inconsistent parenting, reduced warmth, and economic strain (26, 27). Emerging evidence also suggests that preconception paternal alcohol exposure may alter sperm epigenetic profiles, influencing offspring neurodevelopment (28, 29).

However, evidence from non-Western contexts remains sparse, and whether these behavioral, familial, and biological pathways operate similarly in culturally distinct settings is unclear. Cultural norms differ across societies (30), and Western findings may not generalize to Asia. In China, alcohol consumption among men is highly prevalent (national estimate ~62% (31), with higher rates in Western regions (32)), yet empirical research linking paternal drinking to child psychosocial functioning is limited (33). This gap is notable given national policy priorities: the Healthy China Action Plan (2019-2030) emphasizes early childhood development and mental health promotion (34), yet evidence to inform family-centered interventions remains scarce.

Preschool-aged children (3-6 years) are particularly vulnerable due to rapid neurodevelopment (35) and reliance on the family environment during this foundational period. Disruptions during this stage can have cascading developmental effects (36), but few studies have examined paternal drinking patterns among families with preschoolers in China or their relationship to early psychosocial functioning.

To address these gaps, this study examines the association between paternal alcohol consumption and preschoolers’ overall psychosocial difficulties and prosocial behavior in Western China using the Strengths and Difficulties Questionnaire (SDQ) (37).

## Method

### Study design and population

This cross-sectional study, conducted with the Municipal Education Bureau and local schools of a western city, targeted preschool children aged >=3 years and <7 years in the western city, China, from February 28 to March 5, 2025. Researchers employed a stratified sampling strategy, using the 13 districts/counties of the western city as strata, and randomly selected 189 public kindergartens from government-registered lists within each stratum. All children in K1-K3 classes were invited, yielding 25,017 parent-child dyads. Parents or guardians completed a standardized survey, providing data on parental demographics, lifestyles, clinical histories, and children’s perinatal characteristics. Data with 2,408 non-parents excluded first, followed by the exclusion of 1,397 dyads due to missing or invalid age data (child age: n = 736; paternal age: n = 427; maternal age: n = 234). The final sample included 21,212 parent-child dyads, of which 16,139 questionnaires were completed by mothers and 5,073 by fathers. Therefore, paternal alcohol consumption was self-reported in 5,073 cases (23.9%) and reported by mothers in 16,139 cases (76.1%). According to the Yan’an Municipal Statistical Bulletins of National Economic and Social Development (2022-2024), the number of children enrolled in kindergarten levels K1-K3 ranged from 63,000 to 86,900, with an average of approximately 74,000 children. Our survey, conducted in early 2025, included 21,212 children from K1-K3, with one parent completing the questionnaire for each child. This sample represents approximately 28.7% of the preschool population in Yan’an.

### Paternal alcohol intake exposure classification

Paternal alcohol consumption was categorized hierarchically into five groups using combined self-reported paternal data and maternal proxy reports: (1) Nondrinkers, (2) Ex-drinkers (cessation ≥ 6 months), and (3) Current drinkers, subdivided into Light (≤2.86 g/day), Moderate (>2.86-20 g/day), and Heavy (>20 g/day) based on daily ethanol intake. Weekly consumption (g/week) was calculated by aggregating beverage-specific intake (e.g., beer, red or white wine) via a standardized conversion formula and converted to daily intake:


Ethanol (g) = ∑[Volume (ml) × Alcohol percentage × 0.789 g/ml density × Frequency]

Alcohol exposure was analyzed both categorically and continuously. Categorical thresholds were adapted from prior epidemiological studies (38), where 2.86 g/day corresponds to approximately one standard drink per week and distinguishes minimal from more regular consumption. The >20 g/day threshold reflects commonly used boundaries for moderate-to-heavy drinking. Although the reference study included a >40 g/day category, this group was extremely small in our sample, resulting in unstable estimates; therefore, individuals consuming >40 g/day were combined into the >20 g/day category to ensure statistical reliability.

### Outcome variables

Children’s mental health was assessed using the official Chinese simplified version of the SDQ, a validated parent-reported instrument designed for Chinese children aged 3-17 years (37, 39). The SDQ comprises 25 items in total, each rated on a 3−point scale (0 = not true, 1 = somewhat true, 2 = certainly true). The Total Difficulties Score (TDS), the primary outcome measure in this study, is derived from 20 items across four subscales: emotional symptoms (5 items), conduct problems (5 items), hyperactivity/inattention (5 items), and peer relationship problems (5 items), yielding a score range of 0-40. The remaining 5 items form the Prosocial Behavior (PB) subscale, which was analyzed separately as an indicator of positive social functioning, with reverse-coded scores indicating greater prosocial tendencies (higher values). Thus, PB < 6 indicates unusually low prosocial behavior. To enhance clinical interpretability, sensitivity analyses employed two binary classifications based on population-specific thresholds (40, 41): (1) TDS > 14, indicating elevated risk, and (2) reverse-coded PB scores < 6, signifying atypically low prosocial tendencies. Continuous TDS was prioritized as the primary metric for statistical sensitivity.

### Definition of confounding factors

Models were adjusted for child characteristics (age, sex, prematurity, birth order, number of siblings, participation in early education, sleep duration), parental factors (maternal and paternal age, education, employment, smoking status, and maternal alcohol intake), and household variables (annual income, living situation, household registration, marital status). Sociodemographic context was addressed through household registration type (urban, rural, collective, or unregistered), capturing variations in resource access.

For stratified analyses, continuous variables (child age, paternal age, and maternal age) were categorized into tertiles, creating Low, Medium, and High groups based on the 33rd and 67th percentiles. Several categorical variables were recategorized or excluded due to small sample sizes: Collective household (n = 173) and Not yet registered (n = 57) could not be meaningfully combined with Urban/Rural hukou and were therefore excluded from household registration stratification; income categories >100–300 thousand yuan and >300 thousand yuan were merged (n = 232 in the latter); Never married (n = 77) was combined with Widowed/divorced/separated for marital status; the Ex-smoker category in maternal smoking (n = 13) yielded unstable estimates and was not reported in the final results; sleep duration categories of 10–13 hours and >13 hours were combined (n = 74 in the latter).

### Statistical analysis

Analyses used R version 4.4.1. Continuous variables were tested for normality (Shapiro-Wilk test) and reported as means ± SD (normal) or medians [IQR] (non-normal); categorical variables as counts (percentages). Baseline group differences (TDS >14 vs. ≤14; PB <6 vs. ≥6) were assessed using chi-square (categorical) and Kruskal-Wallis (continuous) tests.

Binary outcomes (TDS >14 vs. ≤14; PB <6 vs. ≥6) were analyzed using multivariable logistic regression. Model 1: unadjusted. Model 2: adjusted for child, parental, and household covariates, plus paternal alcohol status (categorical and continuous). Multicollinearity assessed using GVIF (threshold <2; **Supplementary Table 1**). TDS, PB, and four SDQ subscales (Emotional Symptoms, Conduct Problems, Hyperactivity/Inattention, Peer Problems) were analyzed as continuous outcomes using multivariable linear regression with identical adjustments. Benjamini-Hochberg FDR correction applied to subscales (significance: FDR-adjusted p < 0.05). No correction applied to TDS/PB (primary outcomes). Effect modification examined by stratifying paternal alcohol-outcome associations across all covariates. Interaction terms tested via likelihood ratio tests (significance: p < 0.05). Analyses limited to TDS/PB to preserve power. Significant interactions in **Table 1**; complete results in **Supplementary Table 3**. Dose–response relationships were examined for linearity; no significant nonlinear thresholds were identified. Two-sided p < 0.05 was considered statistically significant, except for SDQ subscale analyses where FDR adjusted p < 0.05 was applied.

**Table 1:** Stratified analysis of paternal alcohol consumption and SDQ outcomes in a western Chinese city, 2025.

Variable	*P*-interaction	Ex-drinker	<=2.86 g/day	>2.86, <=20 g/day	>20 g/day
		Adj. OR (95% CI) P-value	Adj. OR (95% CI) P-value	Adj. OR (95% CI) P-value	Adj. OR (95% CI) P-value
TDS>14					
Child sex	0.004				
Boys		1.59 (1.23, 2.04) <0.001	1.18 (1.00, 1.39) 0.054	1.45 (1.28, 1.64) <0.001	1.89 (1.50, 2.36) <0.001
Girls		1.36 (1.02, 1.80) 0.034	1.17 (0.98, 1.39) 0.088	1.05 (0.92, 1.20) 0.43	1.5 (1.16, 1.93) 0.002
Annual family income level	0.013				
<100		1.52 (1.23, 1.87) <0.001	1.16 (1.02, 1.32) 0.023	1.24 (1.13, 1.37) <0.001	1.46 (1.20, 1.77) <0.001
>=100		1.37 (0.86, 2.11) 0.17	1.21 (0.86, 1.69) 0.27	1.3 (1.04, 1.63) 0.023	2.87 (1.99, 4.08) <0.001
Paternal employment status	0.02				
Working		1.42 (1.16, 1.72) <0.001	1.21 (1.06, 1.37) 0.003	1.26 (1.15, 1.38) <0.001	1.75 (1.47, 2.08) <0.001
Not working		2.71 (1.29, 5.72) 0.008	0.75 (0.44, 1.26) 0.29	1.18 (0.81, 1.73) 0.38	1 (0.46, 2.04) >0.99
Maternal Alcohol intake status	0.018				
Nondrinker		1.45 (1.19, 1.76) <0.001	1.21 (1.06, 1.36) 0.003	1.27 (1.16, 1.39) <0.001	1.78 (1.49, 2.12) <0.001
Ex-drinker		–	0.8 (0.28, 2.25) 0.67	0.5 (0.22, 1.09) 0.083	0.54 (0.14, 1.85) 0.34
Current drinker		2.86 (1.03, 7.79) 0.04	0.57 (0.23, 1.33) 0.21	1.08 (0.60, 1.97) 0.81	1.05 (0.44, 2.40) 0.92
SDQ Prosocial Behavior scores <6					
Maternal employment status	0.002				
Working		1.48 (1.23, 1.78) <0.001	1.19 (1.06, 1.34) 0.004	1.14 (1.05, 1.24) 0.002	1.21 (1.02, 1.44) 0.028
Not working		1.16 (0.86, 1.57) 0.34	0.94 (0.80, 1.11) 0.48	1.28 (1.13, 1.45) <0.001	1.62 (1.25, 2.12) <0.001

Models were adjusted for the same covariates as in **Table 3**. Adjusted OR (95% CI) relative to nondrinkers. P-interaction from likelihood ratio tests; *P<0.05. Continuous variables were categorized by tertiles; child age, paternal age, and maternal age were divided into Low, Medium, and High groups based on the 33rd and 67th percentiles. Categories were merged or removed due to small sample sizes: Household registration excluded Collective household (n = 173) and Not yet registered (n = 57); Income combined >100–300 thousand yuan and >300 thousand yuan (n = 232 in the latter); Marital status combined Never married (n = 77) with Widowed/divorced/separated; Maternal smoking: the Ex-smoker category (n = 13) did not yield stable estimates and was therefore not retained in stratified analyses; Sleep duration combined 10–13 hours and >13 hours (n = 74).

CI, confidence interval; CNY, Chinese Yuan; OR, odds ratio; SDQ, Strengths and Difficulties Questionnaire (Official Chinese version); TDS, Total Difficulties Score.

Additional details on variable construction (Method S2) and the full questionnaires, including the alcohol consumption questionnaire and the SDQ instruments (Questionnaire S1 and Questionnaire S2), are available in the **Supplementary Material**.

## Result

### Baseline characteristics

Baseline characteristics of the study cohort (N = 21,212) are presented in **Table 2**, stratified by TDS groups (TDS ≤14: n = 17,283; TDS >14: n = 3,929). Children with TDS >14 were slightly younger, and their parents were marginally younger as well. Boys had a higher percentage of TDS >14 than girls (19.5% vs. 17.5%). Shorter or longer sleep duration was associated with higher TDS, with the highest percentages among children sleeping <8 hours (37.1%) and >13 hours (25.7%), compared with 13.9% for 10-13 hours. Socioeconomic disadvantages were associated with higher TDS: rural household registration, lower parental education, and lower annual household income. Behavioral and family factors also showed clear patterns, with higher TDS >14 among children whose fathers were current drinkers or smokers, whose mothers smoked, and those living in rented or multigenerational households.

**Table 2:** Baseline characteristics of preschool children in a western Chinese city, 2025: comparison by SDQ total difficulties score (>14 vs. ≤14).

Variable	N = 21,212	TDS >14	TDS ≤14	*p*-value
		N = 3,929	N = 17,283	
Child age (years) ^a^	4.81 (0.89)	4.80 (0.90)	4.82 (0.88)	0.231
Child sex				<0.001
Boys	10,995 (51.8%)	2140 (19.5%)	8855 (80.5%)	
Girls	10,217 (48.2%)	1789 (17.5%)	8428 (82.5%)	
Paternal age (years)	35.95 (4.67)	35.79 (4.68)	35.99 (0.0%)	0.058
Maternal age (years)	34.43 (4.49)	34.04 (4.62)	34.52 (0.0%)	<0.001
Male alcohol content	27.66 (52.56)	26.41 (50.52)	65.22 (0.0%)	<0.001
Living situation				<0.001
Owning a home	11,501 (54.2%)	1928 (16.8%)	9573 (83.2%)	
Mortgage housing	3,380 (15.9%)	605 (17.9%)	2775 (82.1%)	
Renting a home	4,360 (20.6%)	944 (21.7%)	3416 (78.3%)	
Living with family or relatives	1,971 (9.3%)	452 (22.9%)	1519 (77.1%)	
Number of children				0.001
1	6,103 (28.8%)	1148 (18.8%)	4955 (81.2%)	
2	12,760 (60.2%)	2292 (18.0%)	10468 (82.0%)	
3	2,085 (9.8%)	421 (20.2%)	1664 (79.8%)	
4	264 (1.2%)	68 (25.8%)	196 (74.2%)	
Ranking of children				<0.001
1	9,155 (43.2%)	1788 (19.5%)	7367 (80.5%)	
2	9,980 (47.0%)	1696 (17.0%)	8284 (83.0%)	
3	1,656 (7.8%)	339 (20.5%)	1317 (79.5%)	
4	421 (2.0%)	106 (25.2%)	315 (74.8%)	
Household registration of children				<0.001
Urban Household	6,851 (32.3%)	1073 (15.7%)	5778 (84.3%)	
Rural Household	14,131 (66.6%)	2813 (19.9%)	11318 (80.1%)	
Collective Household	173 (0.8%)	32 (18.5%)	141 (81.5%)	
Not yet registered	57 (0.3%)	11 (19.3%)	46 (80.7%)	
Paternal alcohol intake status				<0.001
Nondrinker	11,663 (55.0%)	1954 (16.8%)	9709 (83.2%)	
Ex-drinker	751 (3.5%)	174 (23.2%)	577 (76.8%)	
Current <=2.86 g/day	2,245 (10.6%)	449 (20.0%)	1796 (80.0%)	
Current >2.86, <=20 g/day	5,681 (26.8%)	1130 (19.9%)	4551 (80.1%)	
Current >20, <=40 g/day	762 (3.6%)	191 (25.1%)	571 (74.9%)	
Current >40 g/day	110 (0.5%)	31 (28.2%)	79 (71.8%)	
Maternal Alcohol intake status				<0.001
Nondrinker	20,415 (96.2%)	3723 (18.2%)	16692 (81.8%)	
Ex-drinker	242 (1.1%)	75 (31.0%)	167 (69.0%)	
Current drinker	555 (2.6%)	131 (23.6%)	424 (76.4%)	
Paternal education level				<0.001
≤ Junior high school	6,476 (30.5%)	1520 (23.5%)	4956 (76.5%)	
High school/junior college	9,760 (46.0%)	1660 (17.0%)	8100 (83.0%)	
≥Undergraduate degree	4,976 (23.5%)	749 (15.1%)	4227 (84.9%)	
Maternal education level				<0.001
≤ Junior high school	5,889 (27.8%)	1420 (24.1%)	4469 (75.9%)	
High school/junior college	9,219 (43.5%)	1627 (17.6%)	7592 (82.4%)	
Undergraduate degree	6,104 (28.8%)	882 (14.4%)	5222 (85.6%)	
Annual household income (thousands CNY)				<0.001
≤100	17,107 (80.6%)	3376 (19.7%)	13731 (80.3%)	
>100 to 300	3,873 (18.3%)	519 (13.4%)	3354 (86.6%)	
>300	232 (1.1%)	34 (14.7%)	198 (85.3%)	
Paternal employment status				<0.001
Working	20,100 (94.8%)	3625 (18.0%)	16475 (82.0%)	
Not working	1,112 (5.2%)	304 (27.3%)	808 (72.7%)	
Maternal employment status				<0.001
Working	14,651 (69.1%)	2482 (16.9%)	12169 (83.1%)	
Not working	6,561 (30.9%)	1447 (22.1%)	5114 (77.9%)	
Marital status				<0.001
Married/Living with partner	20,637 (97.3%)	3781 (18.3%)	16856 (81.7%)	
Widowed/divorced/separated	498 (2.3%)	121 (24.3%)	377 (75.7%)	
Never married	77 (0.4%)	27 (35.1%)	50 (64.9%)	
Paternal smoking status				<0.001
Never smoke	8,610 (40.6%)	1466 (17.0%)	7144 (83.0%)	
Ex-smoker	461 (2.2%)	97 (21.0%)	364 (79.0%)	
Current Smoker	12,141 (57.2%)	2366 (19.5%)	9775 (80.5%)	
Maternal smoking status				<0.001
Never smoke	21,017 (99.1%)	3868 (18.4%)	17149 (81.6%)	
Ex-smoker	13 (0.1%)	5 (38.5%)	8 (61.5%)	
Current Smoker	182 (0.9%)	56 (30.8%)	126 (69.2%)	
Child Premature				<0.001
Premature Birth	20,239 (95.4%)	3683 (18.2%)	16556 (81.8%)	
Term Birth	867 (4.1%)	215 (24.8%)	652 (75.2%)	
Post-term Birth	106 (0.5%)	31 (29.2%)	75 (70.8%)	
Sleep duration				<0.001
<8 hours,	552 (2.6%)	205 (37.1%)	347 (62.9%)	
8–9 hours	14,549 (68.6%)	2867 (19.7%)	11682 (80.3%)	
10–13 hours	6,037 (28.5%)	838 (13.9%)	5199 (86.1%)	
>13 hours.	74 (0.3%)	19 (25.7%)	55 (74.3%)	
Early education				0.002
Attended systematic early education program	1,921 (9.1%)	347 (18.1%)	1574 (81.9%)	
Attended non-systematic early education activities	4,879 (23.0%)	901 (18.5%)	3978 (81.5%)	
Never attended any early education activities	14412 (67.9%)	2681 (18.6%)	11731 (81.4%)	

Children’s ages were calculated at baseline (February 2025). SDQ Total Difficulties Score (TDS) was categorized as >14 (elevated difficulties) versus ≤14 (typical development). Data are presented as n (%) or mean ± SD. P values were obtained using the Kruskal–Wallis test for continuous non-normal variables and Fisher’s exact test for categorical variables with expected counts <10; *P < 0.05 was considered statistically significant.

CNY, Chinese Yuan; SDQ, Strengths and Difficulties Questionnaire (Chinese version); TDS, Total Difficulties Score; PB, Prosocial Behavior.

Baseline characteristics stratified by prosocial behavior (PB) are shown in **Supplementary Table 2** (PB <6: n = 10,483; PB ≥6: n = 10,729). Children with PB ≥6 were slightly older, more often girls, and more likely to have longer sleep duration. Higher PB scores were also associated with indicators of socioeconomic advantage, including higher maternal education, higher household income, and urban household registration. Children whose fathers were nondrinkers or never smoked, and those who attended systematic early education programs, also showed higher proportions of PB ≥6.

### Dose-dependent effects of paternal alcohol consumption on children’s mental health

Multivariable regression analyses of 21,212 children showed that paternal alcohol consumption was consistently associated with adverse child mental health outcomes. Children of ex-drinkers had the highest risk across measures, while current drinkers also demonstrated elevated odds compared with nondrinkers. Logistic regression results are presented in **Table 3**, and linear regression results in **Table 4**.

**Table 3:** Associations between paternal alcohol consumption and child mental health outcomes (Binary Classifications).

Outcome	Variable	Adjusted OR (95% CI)	P-value
TDS >14	Nondrinker	Ref.	–
	Current Drinker	**1.15(1.04, 1.26)**	0.005
	Ex-drinker	**1.54(1.28, 1.84)**	<0.001
	<=2.86 g/day (~20 g/week)	**1.21(1.07, 1.36)**	0.002
	>2.86, <=20 g/day (~20–140 g/week)	**1.27(1.16, 1.39)**	<0.001
	>20 g/day (~140 g/week)	**1.73(1.46, 2.04)**	<0.001
	Alcohol intake content (per 100 g/week)	**1.2 (1.11–1.29)**	< 0.001
SDQ Prosocial Behavior scores <6	Nondrinker	Ref.	–
	Current Drinker	**1.12(1.04, 1.21)**	0.003
	Ex-drinker	**1.43(1.23, 1.66)**	<0.001
	<=2.86 g/day (~20 g/week)	**1.11(1.01, 1.22)**	0.027
	>2.86, <=20 g/day (~20–140 g/week)	**1.19(1.12, 1.28)**	<0.001
	>20 g/day (~140 g/week)	**1.34(1.16, 1.54)**	<0.001
	Alcohol intake content (per 100 g/week)	**1.01 (0.93–1.09)**	0.01

Adjusted for child-level covariates (age, sex, prematurity, birth order, number of siblings, early education, sleep duration), parental factors (maternal and paternal age, education, employment, smoking, maternal alcohol intake), and household variables (income, living situation, household registration, marital status). Data are presented as adjusted odds ratios (OR) with 95% confidence intervals (CI) from multivariable logistic regression models. Paternal alcohol consumption was modeled as categorical status (nondrinkers [reference], ex-drinkers, current drinkers by dose: ≤2.86 g/day, >2.86–20 g/day, >20 g/day) and continuous intake (per 100 g/week). TDS >14 indicates elevated risk of mental health difficulties; PB <6 indicates atypically low prosocial behavior. Bold values indicate P < 0.05.

CI, confidence interval; OR, odds ratio; SDQ, Strengths and Difficulties Questionnaire (Chinese version); TDS, Total Difficulties Score; PB, Prosocial Behavior.

**Table 4:** Associations between paternal alcohol consumption and SDQ scores (Continuous Outcomes).

Paternal alcohol consumption	Total difficulties β (95% CI), p	Emotional symptoms β (95% CI), *p*, FDR *p*	Conduct problems β (95% CI), *p*, FDR *p*	Hyperactivity β (95% CI), *p*, FDR *p*	Peer problems β (95% CI), *p*, FDR *p*	Prosocial behavior β (95% CI), p
Nondrinker	Ref.	Ref.	Ref.	Ref.	Ref.	Ref.
Current drinker	-0.928 (-2.48, 0.62), 0.24	-0.443 (-1.02, 0.14), 0.13, 0.54	-0.082 (-0.5, 0.34), 0.71, 0.71	-0.17 (-0.67, 0.33), 0.5, 0.67	-0.234 (-0.84, 0.38), 0.45, 0.67	0.059 (-0.61, 0.73), 0.86
Ex-drinker	**0.994 (0.62, 1.37), <0.001**	**0.344 (0.2, 0.48), <0.001, <0.001**	0.049 (-0.05, 0.15), 0.35, 0.35	**0.283 (0.16, 0.4), <0.001, <0.001**	**0.319 (0.17, 0.47), <0.001, <0.001**	**-0.368 (-0.53, -0.21), <0.001**
Alcohol intake (per 100 g/week)	**0.53 (0.2, 0.86), 0.002**	**0.19 (0.07, 0.31), 0.003, 0.011**	0.07 (-0.02, 0.16), 0.12, 0.12	0.09 (-0.02, 0.2), 0.1, 0.12	**0.18 (0.05, 0.31), 0.008, 0.016**	-0.1 (-0.25, 0.04), 0.17

Models were adjusted for the same covariates as in **Table 3**. Data are presented as regression coefficients (β) with 95% confidence intervals (CI) from multivariable linear regression models. Paternal alcohol consumption was modeled both categorically (nondrinkers [reference], ex-drinkers, current drinkers by dose) and continuously (per 100 g/week). Higher scores indicate greater difficulties for TDS and subscales, and greater prosocial tendencies for PB. For the four SDQ subscales, the Benjamini–Hochberg false discovery rate (FDR) correction was applied to control for multiple testing; both raw and FDR-adjusted P values are reported, with FDR-adjusted P < 0.05 considered significant.

CI, confidence interval; β, regression coefficient; SDQ, Strengths and Difficulties Questionnaire (Chinese version); TDS, Total Difficulties Score; PB, Prosocial Behavior.

Bold values indicate P < 0.05.

For elevated total difficulties (TDS >14), ex-drinkers had 54% higher odds compared with nondrinkers (adjusted OR = 1.54, 95% CI: 1.28-1.84, P < 0.001), while current drinkers overall also showed increased odds (adjusted OR = 1.15, 95% CI: 1.04-1.26, P = 0.005). A clear dose-response gradient emerged, with odds ratios increasing from 1.21 for light consumption to 1.73 for heavy consumption (all P < 0.01; see **Table 3**). Each additional 100 g/week of alcohol intake was associated with 20% higher odds (OR = 1.20, 95% CI: 1.11-1.29, P < 0.001) and higher continuous difficulty scores (β = 0.53 per 100 g/week, 95% CI: 0.20-0.86, P = 0.002; see **Table 4**). When analyzed as a continuous outcome, ex-drinkers showed significantly elevated scores (β = 0.99, 95% CI: 0.62-1.37, P < 0.001), while current drinkers as a group showed no overall difference (P = 0.24).

For atypically low prosocial behavior (PB <6), ex-drinkers had 43% higher odds (adjusted OR = 1.43, 95% CI: 1.23-1.66, P < 0.001), while current drinkers showed 12% higher odds (adjusted OR = 1.12, 95% CI: 1.04-1.21, P = 0.003). A dose-response pattern was observed among current drinkers, with odds ratios ranging from 1.11 to 1.34 across consumption levels (see **Table 3**). However, when modeled as a continuous variable, alcohol intake per 100 g/week was not significantly associated with prosocial scores (P = 0.17). In continuous analyses, ex-drinkers had significantly lower prosocial scores (β = -0.37, 95% CI: -0.53 to -0.21, P < 0.001; see **Table 4**), while current drinkers showed no difference (P = 0.86).

To examine whether associations varied across specific domains of child mental health, we analyzed the four SDQ subscales as continuous outcomes with FDR correction for multiple testing. Ex-drinkers showed significantly elevated scores across three of four subscales: Emotional Symptoms (β = 0.34, 95% CI: 0.20-0.48, FDR-adjusted P < 0.001), Hyperactivity/Inattention (β = 0.28, 95% CI: 0.16-0.40, FDR-adjusted P < 0.001), and Peer Problems (β = 0.32, 95% CI: 0.17-0.47, FDR-adjusted P < 0.001), but not Conduct Problems (P = 0.35; see **Table 4**). Continuous alcohol intake (per 100 g/week) showed significant dose-dependent associations with Emotional Symptoms (β = 0.19, 95% CI: 0.07-0.31, FDR-adjusted P = 0.011) and Peer Problems (β = 0.18, 95% CI: 0.05-0.31, FDR-adjusted P = 0.016), but not with Conduct Problems (P = 0.12) or Hyperactivity/Inattention (P = 0.12). Current drinkers as a group showed no significant associations with any subscale after FDR correction.

No significant nonlinear thresholds were identified in dose–response analyses, possibly due to limited data at higher intake levels.

### Stratified analysis

**Figure 1:**
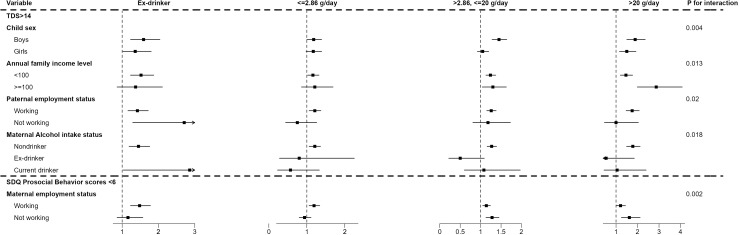
Forest plot of the cross-sectional associations between paternal alcohol consumption and children’s mental health outcomes, stratified by paternal employment status, household income (thousand CNY), child sex, maternal alcohol intake, and maternal employment status. The x-axis displays odds ratios (ORs), with squares and horizontal lines representing point estimates and 95% confidence intervals. P-values correspond to interaction tests for each stratified factor.

After excluding children with collective or unregistered hukou (n = 230), the analytical sample for stratified analyses was N = 20,982. Adjusted ORs and 95% CIs for paternal drinking across subgroups are shown in **Figure 1**, **Table 1**, and **Supplementary Table 3**.

For TDS, four significant effect modifiers were identified: child sex (P−interaction = 0.004), annual family income (P−interaction = 0.013), paternal employment status (P−interaction = 0.020), and maternal alcohol intake (P−interaction = 0.018). Among boys, both moderate and heavy paternal drinking were associated with elevated odds of TDS >14, whereas among girls, only heavy drinking showed a significant association. Higher−income households (≥100,000 CNY/year) showed stronger associations, with heavy drinking linked to nearly threefold increased odds (OR = 2.87, 95% CI: 1.99–4.08), compared with more modest elevations in lower−income households (OR = 1.46, 95% CI: 1.20–1.77). Among children of working fathers, paternal drinking demonstrated clear dose−response patterns; estimates among non−working fathers were imprecise due to small sample size. Maternal alcohol intake further modified the association: dose−response patterns were evident among children of nondrinking mothers, whereas among children of current−drinking mothers, only paternal ex−drinking showed a significant association.

For PB, maternal employment was the only significant effect modifier (P−interaction = 0.002). Among children of working mothers, paternal ex−drinking and all levels of current drinking were associated with higher odds of reduced prosocial behavior. Among children of non−working mothers, moderate−to−heavy paternal drinking showed significant associations, whereas light drinking and ex−drinking did not.

## Discussion

This cross-sectional study of 21,212 preschool children in western China investigated associations between paternal alcohol consumption and child mental health outcomes, adjusting for maternal drinking and other family factors. Three key findings emerged. First, ex-drinkers consistently demonstrated the highest risk across outcomes, with 54% higher odds for total difficulties (TDS >14) and 43% higher odds for reduced prosocial behavior (PB <6). Second, among current drinkers, outcome-specific dose-response patterns were observed. For TDS, odds increased progressively from light to heavy drinking. For PB, categorical analyses suggested slight elevations, but continuous modeling showed only weak associations, reflecting a flat pattern without clear dose-response. Third, significant effect modification was observed by child sex, family income, parental employment, and maternal drinking status (p-interaction = 0.018 for maternal alcohol). Paternal alcohol effects were strongest among boys, higher-income families, and families where mothers abstained from alcohol (97% of sample). Maternal current drinking attenuated paternal effects, though small sample sizes (2.6%) preclude definitive conclusions.

These findings demonstrate independent associations between paternal alcohol consumption and preschool children’s emotional and behavioral outcomes, with effect magnitudes varying across family contexts and maternal drinking patterns.

### Dose-response patterns and interpretive challenges

The prevalence of paternal drinking in our sample (41.5%) was lower than previously reported national estimates (62%) (31) and regional estimates from Western China (77.9%) (32). This difference may reflect lighter drinking patterns among fathers of preschool-aged children. The observed dose-response relationship between paternal alcohol consumption and children’s emotional and behavioral difficulties is consistent with prior evidence linking parental substance use to adverse child outcomes (12). Our finding of higher odds among ex-drinkers should be interpreted with caution due to several methodological limitations. Without data on reasons for cessation, pre-cessation drinking patterns, or paternal health status, we cannot distinguish between potential explanations including health-related cessation, residual confounding (e.g., shared genetic predispositions (6))), or reverse causation (e.g., child behavior prompting abstinence). Prospective cohort studies with detailed information on drinking trajectories and reasons for cessation are needed to clarify these associations.

Beyond these uncertainties, the dose-response pattern for total difficulties suggests that higher levels of paternal alcohol consumption may contribute to children’s emotional and behavioral problems through family and environmental pathways. Recent studies show that paternal drinking is associated with reduced parenting sensitivity, lower family cohesion, impaired parent-child interactions, and harsh parenting, all of which compromise children’s socio-emotional development (42–44). Heavy drinking may create cumulative stress that particularly elevates risk for emotional symptoms and peer problems, consistent with severity-dependent effects documented in longitudinal research (12).

In contrast, reduced prosocial behavior (PB <6) was only modestly associated with paternal alcohol intake, showing a flat pattern without linear dose-response. Evidence indicates that parental alcohol use can disrupt family functioning and social learning processes, limiting opportunities for children to acquire prosocial skills (45, 46). This divergence between outcomes suggests that emotional difficulties and prosocial deficits may reflect different developmental vulnerabilities, though the specific mechanisms underlying these differential patterns require further investigation with direct measures of family processes and parent-child interactions.

### Reconciling categorical vs. continuous findings

An apparent divergence emerged between categorical and continuous models. Categorical analyses suggested that current paternal drinking increased the likelihood of children crossing clinical thresholds for both total difficulties and reduced prosocial behavior, whereas continuous models did not show overall mean differences. This pattern is common in child mental health research, where categorical outcomes capture clinically meaningful impairment while continuous outcomes reflect average distributional shifts (47, 48).

Such divergence likely reflects threshold effects rather than true inconsistency. Paternal alcohol consumption may disproportionately elevate risk among children at the extreme end of the distribution, increasing the proportion who exceed clinical cutoffs without shifting mean scores—a phenomenon also noted in studies of familial risk factors (49). Supporting this interpretation, dose-response analyses showed stronger associations at heavier drinking levels, consistent with prior evidence linking parental alcohol use to adverse child outcomes (12).

Together, these findings highlight the importance of analyzing both categorical and continuous outcomes (50). Categorical models identify clinically significant impairment, while continuous models provide insight into population−level shifts (12). The elevated odds for categorical outcomes despite null continuous associations suggest that paternal drinking primarily confers risk for clinically relevant difficulties rather than subtle mean−level changes.

### Stratified vulnerabilities: sex, socioeconomics, and employment status

Stratified analyses revealed substantial effect modification. Associations between paternal drinking and child outcomes varied by socioeconomic status (family income, parental employment), child sex, and maternal drinking status. In higher-income families [>100,000 CNY/year, exceeding the 2023 average household income of ~94,692 CNY for a three-person family (51)], paternal heavy drinking (>20 g/day) showed stronger associations with total difficulties (OR = 2.99) compared to lower-income families. This pattern may reflect differing alcohol consumption contexts across socioeconomic strata (52), though the specific mechanisms—whether related to drinking patterns, family stress profiles, or other unmeasured factors—remain to be elucidated (53–58). Paternal unemployment (5.2% of sample) was also associated with elevated risk (OR = 2.46 for ex-drinkers), though small sample size limits interpretation. Boys showed consistently higher odds of total difficulties across all paternal drinking levels (OR = 1.88 for >20 g/day) compared to girls, consistent with prior evidence of sex-specific vulnerability to early-life environmental stressors (59–63). The biological and developmental bases for this pattern require further investigation, though sex differences in stress reactivity and neurodevelopmental trajectories have been proposed (60–62). Maternal drinking status significantly modified the association between paternal alcohol and children’s outcomes (p-interaction = 0.018). Paternal effects were most pronounced when mothers were nondrinkers (97% of sample), and attenuated when mothers were current drinkers (n = 555, 2.6%). However, the small number of families with maternal drinking precludes definitive conclusions regarding this pattern. This interaction warrants replication in populations with higher maternal alcohol prevalence. Maternal employment status also modified associations with prosocial behavior (PB <6). Among employed mothers, paternal drinking—including ex-drinking—showed consistent associations with reduced prosocial behavior. In families with non-employed mothers, only heavy paternal drinking (>20 g/day, OR = 1.62) was associated with reduced prosocial behavior, while lower-level exposures showed no significant associations. Whether this pattern reflects differences in parental time availability, family dynamics, or other contextual factors cannot be determined from the current data (64–67).

These stratified findings are exploratory and were not adjusted for multiple comparisons. While they suggest that family socioeconomic context, child characteristics, and parental factors shape vulnerability to paternal alcohol exposure, the mechanisms underlying these interactions remain unclear and require empirical validation in future studies with dedicated measures of potential mediators.

## Conclusion

This cross-sectional study shows that paternal alcohol consumption is associated with emotional and behavioral difficulties in preschool children, after adjusting for maternal drinking and other family factors. Associations showed a dose-response pattern for total difficulties, with stronger effects at higher consumption levels. Ex-drinkers also exhibited elevated odds for both outcomes. Prosocial behavior showed weaker associations with paternal alcohol intake. Effect modification was observed by child sex, family income, parental employment, and maternal drinking status, with strongest associations among boys, higher-income families, and families where mothers abstained from alcohol. These findings highlight the importance of considering paternal alcohol use in family-based child health interventions, with tailored approaches for higher-risk subgroups (e.g., boys, higher-income families). Longitudinal studies with repeated measures of both parental alcohol use and child developmental trajectories are needed to establish temporal relationships and identify critical exposure windows for intervention.

## Limitations

This study investigates paternal alcohol consumption and preschool children’s psychosocial outcomes, yet several limitations should be noted. First, the cross-sectional design precludes causal inference, as temporal ordering between exposure and outcome cannot be established. Second, the study was conducted in Western China, where male drinking prevalence has historically been higher than national averages (77.9% in 2015 (68) vs. 62% (31) nationally). Our 2025 estimate of 41.5% may therefore not generalize to urban regions with different regulatory environments or drinking cultures. Third, paternal alcohol use was self-reported, and in some cases reported by mothers, which may introduce non-differential misclassification; such misclassification typically biases associations toward the null and may underestimate true effect sizes. Fourth, several important family-level confounders—parental mental health, psychiatric history, intimate partner violence, and marital conflict—were not measured. These factors may relate to both paternal drinking and child behavioral outcomes, leading to residual confounding in either direction. Finally, the small number of ex-drinkers (n = 400) limited the precision of estimates for this subgroup. Future research should employ larger and more diverse cohorts, incorporate objective alcohol measures, and use longitudinal designs to clarify temporal relationships and potential mediating pathways such as family dysfunction.

## Data Availability

The raw data supporting the conclusions of this article will be made available by the authors, without undue reservation.
