# Supplementary material for: Paternal drinking in western China is associated with preschool children’s well-being: a cross-sectional SDQ study
Source: Front Psychiatry. 2026 Apr 10;17:1739728. doi: 10.3389/fpsyt.2026.1739728 (PMC13106400; doi:10.3389/fpsyt.2026.1739728)
Supplement: Supplementary file 1 [file Supplementaryfile1.docx]

**Supplement Materials**

**Table S1 Multicollinearity assessment of covariates in SDQ outcome models, western China, 2025**

| Covariate | GVIF | Df | Adjusted GVIF |
| --- | --- | --- | --- |
| Paternal alcohol intake status | 1.760 | 2 | 1.152 |
| Child age (years) | 1.053 | 1 | 1.026 |
| Living situation | 1.131 | 1 | 1.063 |
| Number of children | 2.067 | 1 | 1.438 |
| Ranking of children | 2.222 | 1 | 1.491 |
| Household registration of children | 1.274 | 1 | 1.129 |
| Child sex | 1.011 | 1 | 1.005 |
| Sleep duration | 1.026 | 1 | 1.013 |
| Early education | 1.079 | 1 | 1.039 |
| Paternal education level | 1.796 | 1 | 1.340 |
| Maternal education level | 1.862 | 1 | 1.365 |
| Annual household income | 1.098 | 1 | 1.048 |
| Paternal employment status | 1.117 | 1 | 1.057 |
| Maternal employment status | 1.339 | 1 | 1.157 |
| Marital status | 1.032 | 1 | 1.016 |
| Paternal age (years) | 2.707 | 1 | 1.645 |
| Maternal age (years) | 2.717 | 1 | 1.648 |
| Child Premature | 1.005 | 1 | 1.003 |
| Paternal smoking status | 1.221 | 1 | 1.105 |
| Maternal smoking status | 1.200 | 1 | 1.096 |
| Maternal alcohol intake status | 1.190 | 1 | 1.091 |

**Note:**

Generalized Variance Inflation Factor (GVIF) was assessed for covariates in multivariable logistic regression models of SDQ outcomes (TDS >14, PB <6). GVIF < 2 indicated no evidence of multicollinearity. Adjusted for child-level covariates (age, sex, prematurity, birth order, number of siblings, early education, sleep duration), parental factors (maternal and paternal age, education, employment, smoking, maternal alcohol intake), and household variables (income, living situation, household registration, marital status).

Abbreviations: GVIF = Generalized Variance Inflation Factor; Df = Degrees of Freedom; Adjusted GVIF is calculated as GVIF^1/(2×Df)^ to allow comparison across variables with different degrees of freedom; CNY = Chinese Yuan; SDQ = Strengths and Difficulties Questionnaire (Chinese version); TDS = Total Difficulties Score; PB = Prosocial Behavior.

**Table S2. Baseline characteristics of preschool children in a western Chinese city, 2025: comparison by SDQ Prosocial Behavior Score (PB <6 vs. PB ≥6)**

| **Variable** | **N = 21,212** | **PB<6** | **PB≥6** | **p-value** |
| --- | --- | --- | --- | --- |
|  |  | **N = 10,483** | **N = 10,729** |  |
| **Child age (years) ^a^** | 4.81(0.89) | 4.74(0.91) | 4.9(0.87) | <0.001 |
| **Child sex** |  |  |  | <0.001 |
| Boys | 10995(51.8%) | 5683(51.7%) | 5312(48.3%) |  |
| Girls | 10217(48.2%) | 4800(47.0%) | 5417(53.0%) |  |
| **Paternal age (years)** | 35.95(4.67) | 35.8(4.53) | 36.08(4.77) | <0.001 |
| **Maternal age (years)** | 34.43(4.49) | 34.29(4.42) | 34.57(4.53) | <0.001 |
| **Male alcohol content** | 27.66(52.56) | 29.29(54.7) | 26.06(50.33) | <0.001 |
| **Living situation** |  |  |  | 0.042 |
| Owning a home | 11501(54.2%) | 5624(48.9%) | 5877(51.1%) |  |
| Mortgage housing | 3380(15.9%) | 1655(49.0%) | 1725(51.0%) |  |
| Renting a home | 4360(20.6%) | 2250(51.6%) | 2110(48.4%) |  |
| Living with family or relatives | 1971(9.3%) | 954(48.4%) | 1017(51.6%) |  |
| **Number of children** |  |  |  | 0.561 |
| 1 | 6103(28.8%) | 2974(48.7%) | 3129(51.3%) |  |
| 2 | 12760(60.2%) | 6349(49.8%) | 6411(50.2%) |  |
| 3 | 2085(9.8%) | 1025(49.2%) | 1060(50.8%) |  |
| 4 | 264(1.2%) | 135(51.1%) | 129(48.9%) |  |
| **Ranking of children** |  |  |  | 0.993 |
| 1 | 9155(43.2%) | 4518(49.4%) | 4637(50.7%) |  |
| 2 |  |  |  |  |
| 3 |  |  |  |  |
| 4 |  |  |  |  |
| **Household registration of children** |  |  |  | 0.06 |
| Urban Household | 6851(32.3%) | 3302(48.2%) | 3549(51.8%) |  |
| Rural Household | 14131(66.6%) | 7066(50.0%) | 7065(50.0%) |  |
| Collective Household | 173(0.8%) | 84(48.6%) | 89(51.5%) |  |
| Not yet registered | 57(0.3%) | 31(54.4%) | 26(45.6%) |  |
| **Paternal alcohol intake status** |  |  |  | <0.001 |
| Nondrinker | 11663(55.0%) | 5546(47.6%) | 6117(52.5%) |  |
| Ex-drinker | 751(3.5%) | 419(55.8%) | 332(44.2%) |  |
| Current <=2.86 g/day | 2245(10.6%) | 1122(50.0%) | 1123(50.0%) |  |
| Current >2.86, <=20 g/day | 5681(26.8%) | 2924(51.5%) | 2757(48.5%) |  |
| Current >20, <=40 g/day | 762(3.6%) | 409(53.7%) | 353(46.3%) |  |
| Current >40 g/day | 110(0.5%) | 63(57.3%) | 47(42.7%) |  |
| **Maternal Alcohol intake status** |  |  |  | 0.111 |
| Nondrinker | 20415(96.2%) | 10064(49.3%) | 10351(50.7%) |  |
| Ex-drinker | 242(1.1%) | 121(50.0%) | 121(50.0%) |  |
| Current drinker | 555(2.6%) | 298(53.7%) | 257(46.3%) |  |
| **Paternal education level** |  |  |  | <0.001 |
| ≤ Junior high school | 6476(30.5%) | 3373(52.1%) | 3103(47.9%) |  |
| High school/junior college | 9760(46.0%) | 4691(48.1%) | 5069(51.9%) |  |
| ≥Undergraduate degree | 4976(23.5%) | 2419(48.6%) | 2557(51.4%) |  |
| **Maternal education level** |  |  |  | <0.001 |
| ≤ Junior high school | 5889(27.8%) | 3061(52.0%) | 2828(48.0%) |  |
| High school/junior college | 9219(43.5%) | 4553(49.4%) | 4666(50.6%) |  |
| Undergraduate degree | 6104(28.8%) | 2869(47.0%) | 3235(53.0%) |  |
| **Annual household income (thousands CNY)** |  |  |  | <0.001 |
| ≤100 | 17107(80.6%) | 8628(50.4%) | 8479(49.6%) |  |
| >100 to 300 | 3873(18.3%) | 1772(45.8%) | 2101(54.3%) |  |
| >300 | 232(1.1%) | 83(35.8%) | 149(64.2%) |  |
| **Paternal employment status** |  |  |  | 0.002 |
| Working | 20100(94.8%) | 9885(49.2%) | 10215(50.8%) |  |
| Not working | 1112(5.2%) | 598(53.8%) | 514(46.2%) |  |
| **Maternal employment status** |  |  |  | <0.001 |
| Working | 14651(69.1%) | 7128(48.7%) | 7523(51.4%) |  |
| Not working | 6561(30.9%) | 3355(51.1%) | 3206(48.9%) |  |
| **Marital status** |  |  |  | 0.345 |
| Married/Living with partner | 20637(97.3%) | 10215(49.5%) | 10422(50.5%) |  |
| Widowed/divorced/separated | 498(2.3%) | 231(46.4%) | 267(53.6%) |  |
| Never married | 77(0.4%) | 37(48.1%) | 40(52.0%) |  |
| **Paternal smoking status** |  |  |  | 0.019 |
| Never smoke | 8610(40.6%) | 4179(48.5%) | 4431(51.5%) |  |
| Ex-smoker | 461(2.2%) | 246(53.4%) | 215(46.6%) |  |
| Current Smoker | 12141(57.2%) | 6058(49.9%) | 6083(50.1%) |  |
| **Maternal smoking status** |  |  |  | 0.194 |
| Never smoke | 21017(99.1%) | 10371(49.4%) | 10646(50.7%) |  |
| Ex-smoker | 13(0.1%) | 5(38.5%) | 8(61.5%) |  |
| Current Smoker | 182(0.9%) | 107(58.8%) | 75(41.2%) |  |
| **Child Premature** |  |  |  | 0.006 |
| Premature Birth | 20239(95.4%) | 10049(49.7%) | 10190(50.4%) |  |
| Term Birth | 867(4.1%) | 392(45.2%) | 475(54.8%) |  |
| Post-term Birth | 106(0.5%) | 42(39.6%) | 64(60.4%) |  |
| **Sleep duration** |  |  |  | <0.001 |
| <8 hours, | 552(2.6%) | 362(65.6%) | 190(34.4%) |  |
| 8–9 hours | 14549(68.6%) | 7360(50.6%) | 7189(49.4%) |  |
| 10–13 hours | 6037(28.5%) | 2729(45.2%) | 3308(54.8%) |  |
| >13 hours. | 74(0.3%) | 31(41.9%) | 43(58.1%) |  |
| **Early education** |  |  |  | <0.001 |
| Attended systematic early education program | 1921(9.1%) | 880(45.8%) | 1041(54.2%) |  |
| Attended non-systematic early education activities | 4879(23.0%) | 2332(47.8%) | 2547(52.2%) |  |
| Never attended any early education activities | 14412(67.9%) | 7271(50.5%) | 7141(49.6%) |  |

SDQ Prosocial Behavior (PB) groups: <6 (low prosocial behavior) vs. ≥6 (high prosocial behavior). Data presented as n (%) or mean ± SD.

Abbreviations: CNY = Chinese Yuan; SDQ = Strengths and Difficulties Questionnaire (Chinese version); TDS = Total Difficulties Score.

Notes:

a Children’s ages calculated as of February 2025, at baseline.

P-values from Kruskal-Wallis test (continuous variables, non-normal) or Fisher’s exact test (categorical variables, expected counts <10); **p*<0.05.

Table S3 Additional Stratified Analysis Results for Paternal Alcohol Consumption and TDS

| **Variable** | ***P*-interaction** | **Ex-drinker** | **<=2.86 g/day** | **>2.86, <=20 g/day** | **>20 g/day** |
| --- | --- | --- | --- | --- | --- |
|  |  | **Adj. OR (95% CI) P-value** | **Adj. OR (95% CI) P-value** | **Adj. OR (95% CI) P-value** | **Adj. OR (95% CI) P-value** |
| **TDS>14** |  |  |  |  |  |
| **Child age** | 0.46 |  |  |  |  |
| Low <=4.3 |  | 1.66 (1.21, 2.24), 0.001 | 1.18 (0.96, 1.45), 0.11 | 1.18 (1.01, 1.37), 0.034 | 1.84 (1.35, 2.49), <0.001 |
| Medium >4.3, <= 5.3 |  | 1.37 (0.99, 1.88), 0.054 | 1.09 (0.88, 1.33), 0.44 | 1.27 (1.10, 1.48), 0.001 | 1.46 (1.10, 1.92), 0.008 |
| High <7 |  | 1.45 (1.00, 2.06), 0.045 | 1.29 (1.03, 1.60), 0.026 | 1.32 (1.12, 1.56), <0.001 | 1.89 (1.39, 2.54), <0.001 |
| **Living situation** | 0.26 |  |  |  |  |
| Owning a home |  | 1.36 (1.03, 1.78), 0.026 | 1.14 (0.96, 1.36), 0.14 | 1.3 (1.15, 1.47), <0.001 | 1.66 (1.30, 2.10), <0.001 |
| Mortgage housing |  | 1.08 (0.64, 1.76), 0.75 | 1.39 (1.01, 1.88), 0.037 | 1.13 (0.90, 1.42), 0.28 | 2.22 (1.45, 3.35), <0.001 |
| Renting a home |  | 2.2 (1.49, 3.22), <0.001 | 1.2 (0.93, 1.52), 0.15 | 1.26 (1.04, 1.52), 0.02 | 1.45 (0.99, 2.10), 0.052 |
| Living with family or relatives |  | 1.71 (0.96, 2.96), 0.06 | 1 (0.68, 1.45), >0.99 | 1.28 (0.96, 1.70), 0.092 | 1.96 (1.17, 3.22), 0.009 |
| **Number of children** | 0.79 |  |  |  |  |
| 1 |  | 1.3 (0.92, 1.81), 0.12 | 1.02 (0.81, 1.29), 0.85 | 1.24 (1.05, 1.46), 0.01 | 1.89 (1.35, 2.62), <0.001 |
| 2 |  | 1.53 (1.18, 1.96), 0.001 | 1.24 (1.06, 1.45), 0.007 | 1.3 (1.16, 1.46), <0.001 | 1.74 (1.39, 2.15), <0.001 |
| 3 |  | 2.08 (1.14, 3.70), 0.014 | 0.99 (0.67, 1.43), 0.94 | 1.09 (0.82, 1.44), 0.57 | 1.34 (0.78, 2.25), 0.28 |
| 4 |  | 1.43 (0.23, 6.90), 0.67 | 2.73 (0.92, 8.09), 0.067 | 1.7 (0.67, 4.32), 0.26 | 1.01 (0.19, 4.40), >0.99 |
| **Ranking of children** | 0.73 |  |  |  |  |
| 1 |  | 1.43 (1.08, 1.87), 0.011 | 1.15 (0.96, 1.38), 0.13 | 1.26 (1.10, 1.43), <0.001 | 1.73 (1.33, 2.24), <0.001 |
| 2 |  | 1.47 (1.09, 1.96), 0.011 | 1.19 (0.99, 1.43), 0.058 | 1.29 (1.12, 1.47), <0.001 | 1.79 (1.39, 2.28), <0.001 |
| 3 |  | 2.01 (1.00, 3.92), 0.044 | 0.93 (0.61, 1.41), 0.75 | 1.01 (0.74, 1.39), 0.94 | 1.11 (0.58, 2.03), 0.75 |
| 4 |  | 1.57 (0.40, 5.18), 0.48 | 1.75 (0.81, 3.73), 0.15 | 2.22 (1.16, 4.25), 0.016 | 2.76 (0.82, 8.75), 0.089 |
| **Household registration of children** | 0.65 |  |  |  |  |
| Urban Household |  | 1.82 (1.30, 2.51), <0.001 | 1.19 (0.93, 1.51), 0.17 | 1.38 (1.17, 1.62), <0.001 | 1.92 (1.43, 2.55), <0.001 |
| Rural Household |  | 1.38 (1.09, 1.73), 0.007 | 1.18 (1.02, 1.35), 0.024 | 1.21 (1.09, 1.34), <0.001 | 1.6 (1.29, 1.97), <0.001 |
| **Sleep duration** | 0.093 |  |  |  |  |
| <8 hours, |  | 1.08 (0.42, 2.64), 0.88 | 1.52 (0.83, 2.76), 0.17 | 1.23 (0.75, 2.01), 0.4 | 1.05 (0.40, 2.64), 0.91 |
| 8–9 hours |  | 1.52 (1.21, 1.89), <0.001 | 1.19 (1.03, 1.37), 0.016 | 1.18 (1.07, 1.32), 0.002 | 1.7 (1.39, 2.07), <0.001 |
| >10 hours |  | 1.56 (1.04, 2.29), 0.027 | 1.06 (0.81, 1.37), 0.67 | 1.5 (1.25, 1.80), <0.001 | 1.84 (1.27, 2.61), <0.001 |
| **Early education** | 0.56 |  |  |  |  |
| Attended systematic early education program |  | 1.27 (0.65, 2.34), 0.45 | 1.51 (0.98, 2.29), 0.058 | 1.34 (0.99, 1.82), 0.06 | 1.41 (0.76, 2.49), 0.25 |
| Attended non-systematic early education activities |  | 1.78 (1.24, 2.53), 0.002 | 1.46 (1.13, 1.87), 0.003 | 1.44 (1.20, 1.74), <0.001 | 2.16 (1.51, 3.05), <0.001 |
| Never attended any early education activities |  | 1.41 (1.10, 1.78), 0.005 | 1.06 (0.92, 1.23), 0.41 | 1.19 (1.07, 1.32), 0.002 | 1.63 (1.32, 2.00), <0.001 |
| **Paternal education level** | 0.81 |  |  |  |  |
| ≤ Junior high school |  | 1.75 (1.23, 2.47), 0.002 | 1.16 (0.95, 1.40), 0.14 | 1.22 (1.05, 1.41), 0.009 | 1.62 (1.23, 2.14), <0.001 |
| High school/junior college |  | 1.32 (0.99, 1.74), 0.056 | 1.17 (0.97, 1.40), 0.1 | 1.33 (1.17, 1.52), <0.001 | 1.79 (1.38, 2.31), <0.001 |
| ≥Undergraduate degree |  | 1.64 (1.11, 2.38), 0.01 | 1.22 (0.90, 1.64), 0.19 | 1.19 (0.97, 1.45), 0.093 | 1.69 (1.14, 2.46), 0.007 |
| **Maternal education level** | 0.084 |  |  |  |  |
| ≤ Junior high school |  | 1.3 (0.91, 1.83), 0.14 | 1.18 (0.97, 1.44), 0.1 | 1.26 (1.07, 1.47), 0.004 | 1.39 (1.00, 1.92), 0.043 |
| High school/junior college |  | 1.51 (1.13, 2.00), 0.004 | 1.04 (0.85, 1.26), 0.71 | 1.31 (1.14, 1.50), <0.001 | 1.69 (1.31, 2.18), <0.001 |
| ≥Undergraduate degree |  | 1.77 (1.21, 2.54), 0.002 | 1.39 (1.07, 1.80), 0.013 | 1.17 (0.98, 1.41), 0.086 | 2.25 (1.62, 3.09), <0.001 |
| **Maternal employment status** | 0.96 |  |  |  |  |
| Working |  | 1.48 (1.18, 1.85), <0.001 | 1.21 (1.03, 1.41), 0.017 | 1.27 (1.14, 1.42), <0.001 | 1.75 (1.42, 2.15), <0.001 |
| Not working |  | 1.54 (1.08, 2.15), 0.014 | 1.12 (0.92, 1.37), 0.24 | 1.22 (1.05, 1.42), 0.01 | 1.61 (1.19, 2.14), 0.001 |
| **Marital status** | 0.82 |  |  |  |  |
| Married/Living with partner |  | 1.48 (1.21, 1.79), <0.001 | 1.16 (1.02, 1.31), 0.02 | 1.25 (1.14, 1.37), <0.001 | 1.67 (1.40, 1.99), <0.001 |
| Widowed/divorced/separated/never married |  | 2.3 (0.92, 5.53), 0.067 | 2.41 (1.12, 5.18), 0.024 | 1.5 (0.82, 2.78), 0.19 | 2.27 (0.92, 5.46), 0.071 |
| **Paternal age** | 0.29 |  |  |  |  |
| Low <=33.8 |  | 1.24 (0.86, 1.76), 0.23 | 1.14 (0.92, 1.41), 0.22 | 1.28 (1.10, 1.50), 0.002 | 2.04 (1.53, 2.71), <0.001 |
| Medium >33.8, <=37.3 |  | 1.51 (1.07, 2.11), 0.016 | 1.23 (1.00, 1.51), 0.044 | 1.35 (1.16, 1.56), <0.001 | 1.3 (0.93, 1.77), 0.11 |
| High >37.3 |  | 1.68 (1.24, 2.27), <0.001 | 1.14 (0.92, 1.41), 0.23 | 1.13 (0.96, 1.33), 0.14 | 1.79 (1.34, 2.37), <0.001 |
| **Maternal age** | 0.8 |  |  |  |  |
| Low <=32.5 |  | 1.34 (0.94, 1.89), 0.1 | 1.24 (1.01, 1.51), 0.037 | 1.32 (1.14, 1.53), <0.001 | 1.91 (1.43, 2.54), <0.001 |
| Medium >32.5, <=35.9 |  | 1.51 (1.09, 2.08), 0.012 | 1.15 (0.93, 1.42), 0.2 | 1.32 (1.14, 1.54), <0.001 | 1.52 (1.12, 2.05), 0.006 |
| High <35.9 |  | 1.62 (1.18, 2.20), 0.002 | 1.1 (0.88, 1.37), 0.39 | 1.11 (0.95, 1.31), 0.2 | 1.66 (1.23, 2.22), <0.001 |
| **Child Premature** | 0.41 |  |  |  |  |
| Premature Birth |  | 1.52 (1.25, 1.84), <0.001 | 1.19 (1.05, 1.35), 0.005 | 1.24 (1.13, 1.35), <0.001 | 1.7 (1.43, 2.02), <0.001 |
| Term Birth |  | 0.99 (0.40, 2.28), 0.99 | 0.97 (0.53, 1.72), 0.92 | 1.59 (1.03, 2.44), 0.036 | 1.61 (0.73, 3.40), 0.22 |
| Post-term Birth |  | 3.05 (0.01, 518), 0.68 | 0.47 (0.01, 12.2), 0.68 | 3.04 (0.24, 48.5), 0.39 | 1.7 (0.00, 430), 0.85 |
| **Paternal smoking status** | 0.64 |  |  |  |  |
| Never smoke |  | 1.43 (0.99, 2.01), 0.047 | 1.06 (0.81, 1.38), 0.65 | 1.3 (1.10, 1.54), 0.002 | 1.68 (1.11, 2.48), 0.011 |
| Ex-smoker |  | 1.81 (0.98, 3.41), 0.06 | 0.83 (0.25, 2.42), 0.75 | 0.74 (0.34, 1.54), 0.42 | 0.9 (0.10, 5.50), 0.91 |
| Current Smoker |  | 1.39 (1.07, 1.79), 0.012 | 1.21 (1.05, 1.40), 0.007 | 1.26 (1.13, 1.41), <0.001 | 1.73 (1.42, 2.09), <0.001 |
| **Maternal smoking status** | 0.64 |  |  |  |  |
| Never smoke |  | 1.47 (1.21, 1.77), <0.001 | 1.18 (1.04, 1.33), 0.009 | 1.25 (1.14, 1.36), <0.001 | 1.68 (1.42, 1.99), <0.001 |
| Current Smoker |  | 7.99 (0.94, 69.3), 0.053 | 0.9 (0.11, 6.77), 0.92 | 2.72 (0.44, 16.9), 0.28 | 1.33 (0.02, 65.0), 0.88 |
| **SDQ Prosocial Behavior scores <6** |  |  |  |  |  |
| **Child age** | 0.85 |  |  |  |  |
| Low <=4.3 |  | 1.42 (1.08, 1.87), 0.013 | 1.1 (0.93, 1.29), 0.27 | 1.18 (1.05, 1.33), 0.007 | 1.46 (1.11, 1.93), 0.007 |
| Medium >4.3, <= 5.3 |  | 1.21 (0.94, 1.56), 0.14 | 1.07 (0.91, 1.25), 0.4 | 1.14 (1.02, 1.28), 0.02 | 1.35 (1.07, 1.70), 0.011 |
| High <7 |  | 1.6 (1.19, 2.16), 0.002 | 1.13 (0.95, 1.34), 0.18 | 1.25 (1.10, 1.42), <0.001 | 1.26 (0.98, 1.62), 0.072 |
| **Living situation** | 0.25 |  |  |  |  |
| Owning a home |  | 1.35 (1.09, 1.67), 0.006 | 1.12 (0.98, 1.27), 0.095 | 1.15 (1.04, 1.26), 0.004 | 1.17 (0.96, 1.42), 0.11 |
| Mortgage housing |  | 1.59 (1.07, 2.37), 0.023 | 1.18 (0.92, 1.51), 0.19 | 1.29 (1.08, 1.53), 0.004 | 2.16 (1.48, 3.17), <0.001 |
| Renting a home |  | 1.55 (1.08, 2.24), 0.02 | 1.1 (0.90, 1.35), 0.36 | 1.17 (1.00, 1.37), 0.055 | 1.23 (0.89, 1.71), 0.22 |
| Living with family or relatives |  | 1.06 (0.64, 1.74), 0.82 | 0.87 (0.64, 1.19), 0.39 | 1.25 (0.98, 1.58), 0.068 | 1.41 (0.89, 2.22), 0.14 |
| **Number of children** | 0.16 |  |  |  |  |
| 1 |  | 1.48 (1.12, 1.95), 0.006 | 1.18 (0.98, 1.41), 0.08 | 1.1 (0.97, 1.25), 0.14 | 1.35 (1.01, 1.81), 0.042 |
| 2 |  | 1.27 (1.03, 1.57), 0.026 | 1.06 (0.93, 1.19), 0.38 | 1.24 (1.14, 1.36), <0.001 | 1.26 (1.05, 1.51), 0.012 |
| 3 |  | 1.68 (1.00, 2.84), 0.05 | 1.1 (0.82, 1.48), 0.54 | 1.16 (0.93, 1.45), 0.18 | 1.5 (0.97, 2.34), 0.069 |
| 4 |  | 2.45 (0.62, 11.1), 0.21 | 1.34 (0.51, 3.58), 0.55 | 1.05 (0.48, 2.31), 0.91 | 8.06 (1.92, 46.9), 0.009 |
| **Ranking of children** | 0.56 |  |  |  |  |
| 1 |  | 1.56 (1.24, 1.97), <0.001 | 1.17 (1.01, 1.35), 0.039 | 1.17 (1.05, 1.30), 0.004 | 1.33 (1.06, 1.67), 0.016 |
| 2 |  | 1.23 (0.97, 1.57), 0.083 | 1.02 (0.89, 1.17), 0.8 | 1.23 (1.11, 1.36), <0.001 | 1.3 (1.06, 1.59), 0.012 |
| 3 |  | 1.37 (0.75, 2.54), 0.31 | 1.14 (0.82, 1.59), 0.44 | 1.09 (0.85, 1.39), 0.5 | 1.6 (0.96, 2.68), 0.073 |
| 4 |  | 1.3 (0.46, 3.67), 0.61 | 1.47 (0.75, 2.88), 0.26 | 1.21 (0.69, 2.11), 0.51 | 2.36 (0.79, 7.73), 0.13 |
| **Household registration of children** | 0.17 |  |  |  |  |
| Urban Household |  | 1.61 (1.24, 2.11), <0.001 | 1.22 (1.02, 1.45), 0.029 | 1.18 (1.05, 1.33), 0.005 | 1.54 (1.21, 1.95), <0.001 |
| Rural Household |  | 1.28 (1.05, 1.55), 0.015 | 1.04 (0.93, 1.17), 0.45 | 1.18 (1.09, 1.29), <0.001 | 1.22 (1.02, 1.47), 0.028 |
| **Child sex** | 0.1 |  |  |  |  |
| Boys |  | 1.44 (1.16, 1.79), 0.001 | 0.99 (0.87, 1.13), 0.91 | 1.19 (1.08, 1.31), <0.001 | 1.43 (1.17, 1.74), <0.001 |
| Girls |  | 1.3 (1.04, 1.64), 0.023 | 1.23 (1.07, 1.41), 0.003 | 1.18 (1.07, 1.31), <0.001 | 1.23 (1.00, 1.52), 0.055 |
| **Sleep duration** | 0.85 |  |  |  |  |
| <8 hours, |  | 1.49 (0.58, 4.10), 0.42 | 1.41 (0.76, 2.67), 0.28 | 1.62 (0.97, 2.75), 0.068 | 1.47 (0.58, 3.88), 0.42 |
| 8–9 hours |  | 1.34 (1.11, 1.62), 0.003 | 1.13 (1.01, 1.26), 0.039 | 1.19 (1.09, 1.29), <0.001 | 1.26 (1.06, 1.49), 0.009 |
| >10 hours |  | 1.51 (1.12, 2.03), 0.007 | 1.01 (0.84, 1.21), 0.9 | 1.16 (1.02, 1.32), 0.023 | 1.6 (1.20, 2.13), 0.001 |
| **Early education** | 0.83 |  |  |  |  |
| Attended systematic early education program |  | 1.74 (1.07, 2.87), 0.028 | 1.14 (0.81, 1.61), 0.44 | 1.13 (0.90, 1.43), 0.3 | 1.23 (0.78, 1.94), 0.38 |
| Attended non-systematic early education activities |  | 1.4 (1.04, 1.89), 0.026 | 1.1 (0.90, 1.34), 0.36 | 1.32 (1.14, 1.52), <0.001 | 1.44 (1.07, 1.94), 0.016 |
| Never attended any early education activities |  | 1.31 (1.07, 1.61), 0.008 | 1.09 (0.97, 1.22), 0.15 | 1.15 (1.06, 1.25), 0.001 | 1.32 (1.11, 1.58), 0.002 |
| **Paternal education level** | 0.13 |  |  |  |  |
| ≤ Junior high school |  | 1.02 (0.74, 1.40), 0.92 | 1.04 (0.88, 1.22), 0.65 | 1.09 (0.96, 1.23), 0.19 | 1.35 (1.05, 1.74), 0.021 |
| High school/junior college |  | 1.52 (1.21, 1.92), <0.001 | 1.01 (0.88, 1.17), 0.84 | 1.22 (1.10, 1.35), <0.001 | 1.21 (0.98, 1.50), 0.082 |
| ≥Undergraduate degree |  | 1.57 (1.17, 2.12), 0.003 | 1.47 (1.18, 1.83), <0.001 | 1.27 (1.10, 1.46), 0.001 | 1.6 (1.18, 2.17), 0.002 |
| **Maternal education level** | 0.31 |  |  |  |  |
| ≤ Junior high school |  | 1.09 (0.81, 1.48), 0.56 | 0.99 (0.84, 1.17), 0.91 | 1.16 (1.01, 1.32), 0.035 | 1.3 (0.97, 1.74), 0.078 |
| High school/junior college |  | 1.38 (1.08, 1.75), 0.009 | 1.07 (0.93, 1.24), 0.33 | 1.19 (1.07, 1.32), 0.001 | 1.24 (1.00, 1.54), 0.047 |
| ≥Undergraduate degree |  | 1.78 (1.33, 2.39), <0.001 | 1.25 (1.03, 1.52), 0.021 | 1.2 (1.06, 1.37), 0.004 | 1.53 (1.17, 2.00), 0.002 |
| **Annual family income level** | 0.79 |  |  |  |  |
| <100 |  | 1.34 (1.12, 1.61), 0.001 | 1.09 (0.98, 1.21), 0.11 | 1.17 (1.09, 1.27), <0.001 | 1.28 (1.08, 1.50), 0.003 |
| >=100 |  | 1.52 (1.10, 2.10), 0.012 | 1.16 (0.92, 1.46), 0.22 | 1.23 (1.05, 1.43), 0.009 | 1.53 (1.13, 2.08), 0.006 |
| **Paternal employment status** | 0.71 |  |  |  |  |
| Working |  | 1.39 (1.19, 1.64), <0.001 | 1.11 (1.01, 1.23), 0.032 | 1.18 (1.10, 1.27), <0.001 | 1.33 (1.15, 1.54), <0.001 |
| Not working |  | 1.19 (0.57, 2.50), 0.65 | 0.84 (0.54, 1.29), 0.42 | 1.28 (0.91, 1.81), 0.16 | 1.39 (0.71, 2.80), 0.34 |
| **Marital status** | 0.26 |  |  |  |  |
| Married/Living with partner |  | 1.4 (1.19, 1.64), <0.001 | 1.11 (1.01, 1.22), 0.033 | 1.2 (1.12, 1.28), <0.001 | 1.33 (1.15, 1.54), <0.001 |
| Widowed/divorced/separated/ never married |  | 1.14 (0.52, 2.48), 0.75 | 0.61 (0.31, 1.20), 0.16 | 0.72 (0.42, 1.21), 0.21 | 1.22 (0.54, 2.80), 0.63 |
| **Paternal age** | 0.4 |  |  |  |  |
| Low <=33.8 |  | 1.32 (0.99, 1.75), 0.057 | 1.09 (0.92, 1.28), 0.33 | 1.2 (1.06, 1.35), 0.003 | 1.65 (1.29, 2.13), <0.001 |
| Medium >33.8, <=37.3 |  | 1.31 (0.98, 1.74), 0.066 | 1.06 (0.90, 1.24), 0.51 | 1.14 (1.02, 1.29), 0.025 | 1.32 (1.02, 1.71), 0.038 |
| High >37.3 |  | 1.49 (1.16, 1.93), 0.002 | 1.15 (0.97, 1.36), 0.1 | 1.22 (1.08, 1.38), 0.001 | 1.1 (0.86, 1.40), 0.44 |
| **Maternal age** | 0.38 |  |  |  |  |
| Low <=32.5 |  | 1.35 (1.00, 1.82), 0.049 | 1.07 (0.91, 1.26), 0.39 | 1.11 (0.98, 1.25), 0.09 | 1.63 (1.26, 2.13), <0.001 |
| Medium >32.5, <=35.9 |  | 1.39 (1.06, 1.83), 0.016 | 1.17 (1.00, 1.38), 0.054 | 1.27 (1.13, 1.42), <0.001 | 1.41 (1.10, 1.80), 0.007 |
| High <35.9 |  | 1.44 (1.11, 1.86), 0.006 | 1.04 (0.87, 1.23), 0.68 | 1.18 (1.04, 1.33), 0.008 | 1.05 (0.82, 1.35), 0.68 |
| **Child Premature** | 0.59 |  |  |  |  |
| Premature Birth |  | 1.36 (1.15, 1.60), <0.001 | 1.11 (1.00, 1.22), 0.042 | 1.18 (1.10, 1.27), <0.001 | 1.31 (1.13, 1.51), <0.001 |
| Term Birth |  | 1.65 (0.77, 3.67), 0.2 | 0.89 (0.55, 1.45), 0.63 | 1.21 (0.83, 1.75), 0.32 | 1.85 (0.92, 3.87), 0.091 |
| Post-term Birth |  | 43 (0.81, 5,554), 0.085 | 3.76 (0.31, 59.8), 0.31 | 3.06 (0.51, 21.3), 0.23 | 3.18 (0.03, 145), 0.55 |
| **Paternal smoking status** | 0.56 |  |  |  |  |
| Never smoke |  | 1.55 (1.16, 2.07), 0.003 | 1.2 (0.98, 1.47), 0.073 | 1.27 (1.11, 1.44), <0.001 | 1.65 (1.19, 2.31), 0.003 |
| Ex-smoker |  | 1.48 (0.88, 2.49), 0.14 | 1.55 (0.71, 3.47), 0.27 | 1.54 (0.87, 2.73), 0.14 | 0.87 (0.18, 3.85), 0.85 |
| Current Smoker |  | 1.34 (1.08, 1.66), 0.007 | 1.05 (0.94, 1.17), 0.41 | 1.14 (1.05, 1.24), 0.002 | 1.26 (1.07, 1.48), 0.005 |
| **Maternal smoking status** | 0.48 |  |  |  |  |
| Never smoke |  | 1.37 (1.17, 1.60), <0.001 | 1.09 (1.0, 1.20), 0.064 | 1.18 (1.10, 1.27), <0.001 | 1.32 (1.14, 1.53), <0.001 |
| Current Smoker |  | 3.51 (0.45, 75.4), 0.29 | 2.42 (0.39, 16.4), 0.35 | 1.03 (0.24, 4.29), 0.97 | 3.73 (0.23, 125), 0.38 |
| **Maternal Alcohol intake status** | 0.12 |  |  |  |  |
| Nondrinker |  | 1.37 (1.17, 1.61), <0.001 | 1.08 (0.98, 1.19), 0.1 | 1.19 (1.11, 1.28), <0.001 | 1.36 (1.17, 1.58), <0.001 |
| Ex-drinker |  | (), | 0.48 (0.17, 1.32), 0.16 | 0.57 (0.27, 1.19), 0.14 | 0.44 (0.14, 1.38), 0.16 |
| Current drinker |  | 2.03 (0.79, 5.50), 0.15 | 2.24 (1.15, 4.46), 0.02 | 1.41 (0.86, 2.31), 0.17 | 1.4 (0.70, 2.81), 0.34 |

**Notes:**

Models were adjusted for the same covariates as in Table 2. Adjusted OR (95% CI) relative to nondrinkers. P-interaction from likelihood ratio tests; *P<0.05. Continuous variables were categorized by tertiles; child age, paternal age, and maternal age were divided into Low, Medium, and High groups based on the 33rd and 67th percentiles. Categories were merged or removed due to small sample sizes: **Household registration** excluded *Collective household* (n = 173) and *Not yet registered* (n = 57); **Income** combined *>100–300 thousand yuan* and *>300 thousand yuan* (n = 232 in the latter); **Marital status** combined *Never married* (n = 77) with *Widowed/divorced/separated*; **Maternal smoking**: the *Ex‑smoker* category (n = 13) did not yield stable estimates and was therefore not retained in stratified analyses; **Sleep duration** combined *10–13 hours* and *>13 hours* (n = 74).

Abbreviations: CI = confidence interval; CNY = Chinese Yuan; OR = odds ratio; SDQ = Strengths and Difficulties Questionnaire (Official Chinese version); TDS = Total Difficulties Score.

**Questionnaire S1 The full alcohol consumption questionnaire**

1. Have you ever consumed alcohol? [Single choice]

Yes

No

2. What types of alcohol did you usually drink in the past? [Multiple choice] Dependent on Q1 = Yes

Baijiu (Chinese distilled liquor)

Red wine

Beer

Medicinal liquor

Foreign liquor (e.g., whisky, vodka, rum, gin, brandy)

3. How often did you drink alcohol in the past? [Single choice] Dependent on Q1 = Yes

Once per month

Twice per month

Three times per month

Four times per month

Twice per week

Three times per week

Four times per week

Five times per week

Six times per week

Seven times per week

4. How much did you usually drink each time in the past? [Multiple choice] Dependent on Q1 = Yes

Baijiu: ___ liang (50 g per liang)

Other alcohol: ___ ml

5. Do you currently consume alcohol in the past 6 months? [Single choice]

Yes

No

6. What types of alcohol do you usually drink now? [Multiple choice] Dependent on Q5 = Yes

Baijiu

Red wine

Beer

Medicinal liquor

Foreign liquor (e.g., whisky, vodka, rum, gin, brandy)

7. How often do you currently drink alcohol? [Single choice] Dependent on Q5 = Yes

Once per month

Twice per month

Three times per month

Four times per month

Twice per week

Three times per week

Four times per week

Five times per week

Six times per week

Seven times per week

8. How much do you usually drink each time now? [Multiple choice] Dependent on Q5 = Yes

Baijiu: ___ liang (50 g per liang)

Other alcohol: ___ ml

9. Have your spouse ever consumed alcohol? [Single choice]

Yes

No

10. What types of alcohol did your spouse usually drink in the past? [Multiple choice] Dependent on Q1 = Yes

Baijiu (Chinese distilled liquor)

Red wine

Beer

Medicinal liquor

Foreign liquor (e.g., whisky, vodka, rum, gin, brandy)

11. How often did your spouse drink alcohol in the past? [Single choice] Dependent on Q1 = Yes

Once per month

Twice per month

Three times per month

Four times per month

Twice per week

Three times per week

Four times per week

Five times per week

Six times per week

Seven times per week

12. How much did your spouse usually drink each time in the past? [Multiple choice] Dependent on Q1 = Yes

Baijiu: ___ liang (50 g per liang)

Other alcohol: ___ ml

13. Do your spouse currently consume alcohol in the past 6 months? [Single choice]

Yes

No

14. What types of alcohol do your spouse usually drink now? [Multiple choice] Dependent on Q5 = Yes

Baijiu

Red wine

Beer

Medicinal liquor

Foreign liquor (e.g., whisky, vodka, rum, gin, brandy)

15. How often do your spouse currently drink alcohol? [Single choice] Dependent on Q5 = Yes

Once per month

Twice per month

Three times per month

Four times per month

Twice per week

Three times per week

Four times per week

Five times per week

Six times per week

Seven times per week

16. How much do your spouse usually drink each time now? [Multiple choice] Dependent on Q5 = Yes

Baijiu: ___ liang (50 g per liang)

Other alcohol: ___ ml

**Questionnaire S2 Strengths and Difficulties Questionnaire (SDQ)**

The SDQ used in this study is the official Chinese (Simplified) version for parents of 4–17 year olds, as provided on the SDQ website (https://www.sdqinfo.org). For transparency, the official version has been included as an image in the Supplementary Material.The official English version of the parent-report SDQ (for 4–17 year olds) is also provided below, as available on the SDQ website.


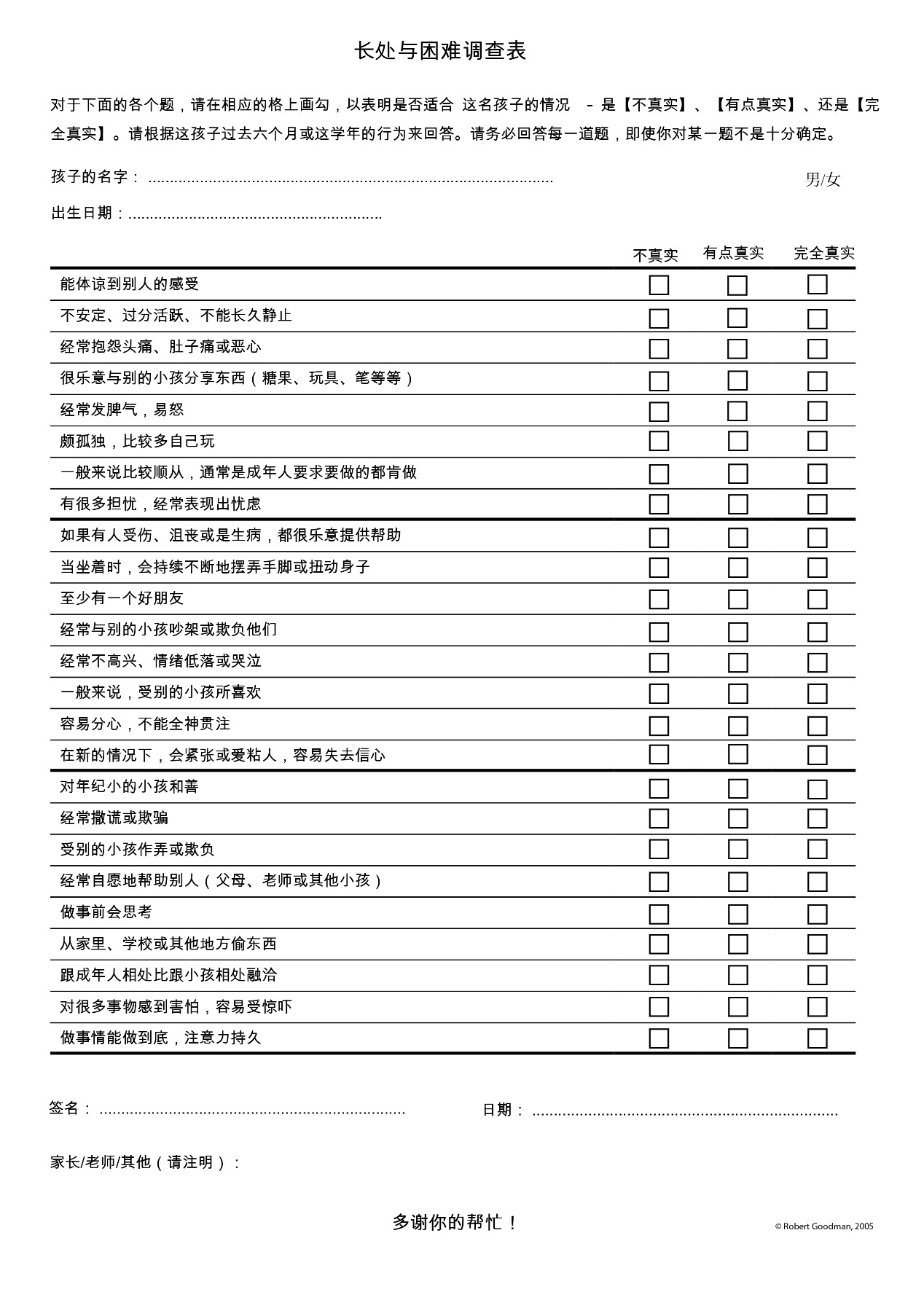


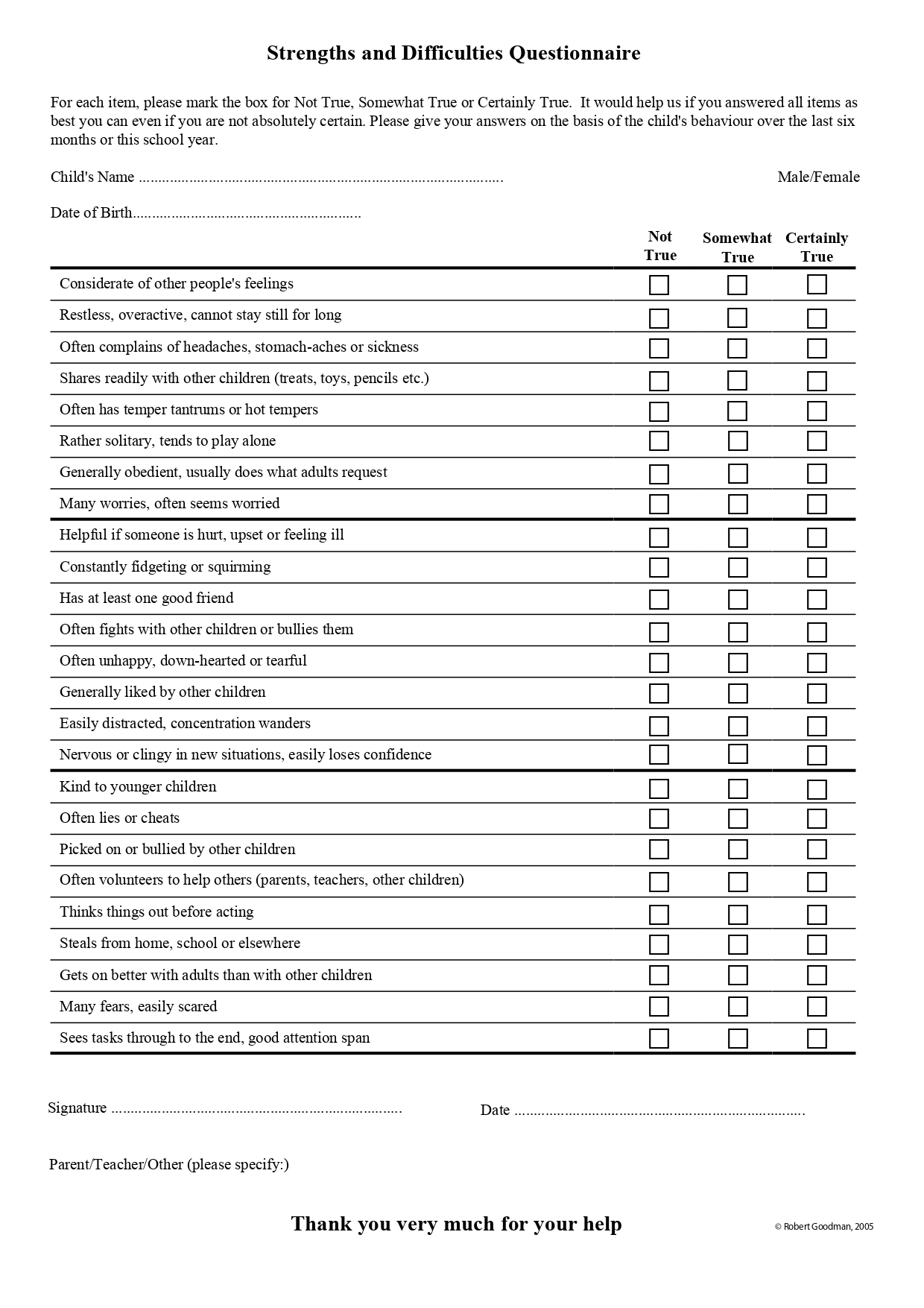


**Method S1 Parental Alcohol Consumption Assessment**

1. **Parental Drinking Status Classification**

Parents (fathers and mothers) were classified into three categories based on self‑reported drinking behavior:

- Nondrinker: Never consumed alcohol.
- Ex‑drinker: Previously consumed alcohol but not in the past 6 months.
- Current drinker: Any alcohol consumption within the past 6 months.

*Note: We defined current drinking as any alcohol consumption within the past 6 months, consistent with our definition of ex‑drinker (cessation ≥6 months). This broader window was chosen to capture low‑frequency drinkers and ensure consistency across parental classifications.*

1. **Weekly Ethanol Intake Estimation (Fathers only)**

For former and current drinkers, weekly ethanol intake was estimated based on beverage type, typical volume per occasion, alcohol concentration, and drinking frequency. Standard ethanol content per drinking occasion was calculated using the formula:

**Ethanol (g) = Σ [Volume (mL) × Alcohol percentage × 0.789 g/mL density × Frequency].**

Standard assumptions for common beverage types were based on typical serving sizes reported in national dietary surveys:

- Baijiu (Chinese distilled liquor): 50 ml × 40% × 0.789 g/ml = 15.8 g ethanol
- Red wine: 100 ml × 13% × 0.789 g/ml = 10.3 g ethanol
- Beer: 500 ml × 4.5% × 0.789 g/ml = 17.8 g ethanol
- Medicinal liquor: 50 ml × 40% × 0.789 g/ml = 15.8 g ethanol
- Foreign spirits (e.g., whisky, vodka): 50 ml × 40% × 0.789 g/ml = 15.8 g ethanol

Weekly intake was calculated as ethanol per occasion × reported weekly frequency. When multiple beverage types were reported, ethanol intake was calculated separately for each type and then summed to obtain total weekly intake.

1. **Drinking Level Classification (Fathers only)**

Daily average ethanol intake (grams of ethanol per day, g/day) was calculated by dividing weekly intake by 7. Drinking levels were classified according to prior epidemiological studies [1], consistent with Chinese Dietary Guidelines (2022) and WHO recommendations

- Light: ≤2.86 g/day
- Moderate: >2.86 to 20 g/day
- Heavy: >20 to 40 g/day
- Very heavy: >40 g/day

*Note: In our sample, very few fathers exceeded 40 g/day of ethanol intake. Consequently, the “Very heavy” category contained insufficient cases for robust statistical analysis. This limitation is acknowledged in the interpretation of results.*

**Method S2 Variable Definitions**

**Child age:** Calculated in years from date of birth to the survey date (2025).

**Birth order:** Child's position among siblings, categorized as first, second, third, or fourth and above.

**Parental age:** Age of mother/father in years at the time of survey completion.

**Educational level:** Highest education completed, categorized as ≤ Junior high school; High school/junior college; ≥ Undergraduate degree.

**Smoking status:** Self-reported smoking behavior categorized as: Never smoker (never smoked or smoked fewer than 100 cigarettes in lifetime); Ex-smoker (formerly smoked regularly but quit); Current smoker (currently smoking cigarettes, regardless of frequency).

**Marital status:** Current relationship status categorized as: Married/Living with partner (legally married or currently cohabiting with a partner); Widowed/divorced/separated (previously married but currently without a partner due to widowhood, divorce, or separation); Never married (never legally married and not currently cohabiting with a partner).

**Annual household income:** Total family income in the past year, categorized as: <100,000 CNY; 100,001–300,000 CNY; >300,000 CNY.

**Number of children:** Total number of children currently living in the household.

**Early education participation:** Early education participation was assessed by asking parents whether their child had attended extracurricular early childhood education programs outside of regular kindergarten, and if so, whether the participation was systematic (regular enrollment in structured programs) or non-systematic (occasional or informal activities). This variable reflects additional educational investment beyond compulsory kindergarten attendance, which is common in urban China.

**Housing stability:** Current living arrangement categorized as: Owning a home (full ownership); Mortgage housing (home ownership with outstanding loan); Renting a home; Living with family/relatives.

**Household registration (Hukou) type:** Official household registration status categorized as: Urban (registered in a city hukou system), Rural (registered in a rural hukou system), Collective (institutional registration such as school, workplace, or government unit), or Not yet registered (children without formal hukou at the time of survey).

**Sleep duration:** Average daily sleep hours reported by parents, categorized as <8 hours, 8–9 hours, 10–13 hours, and >13 hours.

**Child Premature:** Defined by gestational age: premature birth (<37 weeks), term birth (37–41 weeks), and post‑term birth (≥42 weeks).

1. Ortolá, R., et al., *Alcohol Consumption Patterns and Mortality Among Older Adults With Health-Related or Socioeconomic Risk Factors.* JAMA Netw Open, 2024. **7**(8): p. e2424495.
